# Optimizing syphilis screening in South Africa: efficacy of the iStatis antibody test in point-of-care settings amid reinfection challenges

**DOI:** 10.1186/s12981-025-00773-1

**Published:** 2025-08-21

**Authors:** Sharana Mahomed, Savathree Madurai, Someshni Nair, Cherie Cawood, Joshua Eades, Dan Wang, Annalakshmi Subramanian

**Affiliations:** 1 Centre for the AIDS Programme of Research in South Africa, CAPRISA, Durban, South Africa; 2 Department of Medical Microbiology, University of Kwazulu-Natal, Durban, South Africa; 3 Universal Pathology Laboratory, Prospecton, Durban, South Africa; 4 Epicentre Health Research Pty (Ltd), Hillcrest, South Africa; 5 bioLytical Laboratories Inc, Richmond, Canada

**Keywords:** Point-of-Care, Reinfection, Screening, Syphilis, Testing

## Abstract

**Background:**

Syphilis poses a significant threat to global health, particularly in high-risk populations and resource-limited settings. Despite progress in HIV screening, syphilis testing often lags, exacerbating disparities in healthcare delivery. This study evaluated the clinical performance of the iStatis Syphilis Antibody (Ab) Test in South African point-of-care environments.

**Methods:**

A prospective cross-sectional study was conducted with 1,500 enrolled participants across three urban South African sites. The clinical performance of the iStatis Syphilis Antibody (Ab) Test was evaluated using three sample types: capillary blood, EDTA venous whole blood, and plasma. Diagnostic sensitivity and specificity were assessed.

**Results:**

The iStatis test demonstrated excellent diagnostic performance, with sensitivities of 96.40% (capillary blood), 98.80% (venous whole blood), and 99.00% (plasma), and a specificity of 100% across all sample types. A high prevalence of syphilis (33%) was identified. Notably, 90.51% of positive cases were female, and 75.7% of these women were pregnant, highlighting a vulnerable population. The study also revealed a high reinfection rate, suggesting that syphilis can recur relatively quickly post-treatment, underscoring the ongoing transmission challenge.

**Conclusion:**

The iStatis Syphilis Antibody Test is a highly accurate and versatile tool that detects syphilis at different stages using various sample types, making it ideal for use in settings without full laboratory access. The study highlights its potential to improve early diagnosis and control of syphilis, especially in light of high reinfection rates in South Africa. Further research is needed to assess its use in rural areas, long-term performance, and cost-effectiveness to support wider adoption.

## Introduction

Syphilis, a sexually transmitted infection caused by *Treponema pallidum* (TP), remains a public health challenge in South Africa (SA) despite earlier declines in prevalence. Following the widespread introduction of penicillin, antenatal syphilis rates in many countries fell below 1% by the 1960s; however, in parts of sub-Saharan Africa, including SA, prevalence remained higher for decades [1]. For example, SA saw antenatal syphilis seroprevalence drop from 11.2% in 1997 to 1.5% in 2010 [2], attributed largely to routine screening and treatment of pregnant women and syndromic management of genital ulcers [2]. Yet, syphilis persists in diverse populations and has shown resurgent trends in some groups. Notably, the highest burden historically has been in women of reproductive age (detected via antenatal surveys), but recent outbreaks among men (especially men who have sex with men [MSM]) and increases in female cases have been reported globally [3]. Human Immunodeficiency Virus (HIV) co-infection is an additional concern: syphilis prevalence is significantly elevated in people living with HIV [4], and co-infections exacerbate risks of adverse outcomes such as neurosyphilis and increased HIV transmission.

Screening and early diagnosis of syphilis are critical, particularly to prevent mother-to-child transmission and adverse pregnancy outcomes. The World Health Organization (WHO) recommends that all pregnant women be screened for syphilis early in pregnancy, even in low-prevalence settings [5]. Traditional syphilis diagnosis relies on a combination of serological tests. Non-treponemal tests (e.g., Rapid Plasma Reagin [RPR] or Venereal Disease Research Laboratory [VDRL]) detect cardiolipin antibodies that indicate active infection activity, while treponemal tests (e.g., TP particle agglutination [TPPA] or hemagglutination [TPHA], fluorescent treponemal antibody, and enzyme immunoassays) detect antibodies specific to *T. pallidum* [2]. SA algorithms traditionally used RPR for screening, with positives confirmed by a treponemal test, because RPR titers correlate with disease activity and decline after treatment [2]. However, RPR tests require laboratory infrastructure and can yield false positives in patients with other conditions [2] or false negatives due to the *prozone phenomenon* in cases of very high antibody titers [6]. The prozone effect (seen especially in secondary syphilis and HIV co-infection) can cause a false-negative non-treponemal result because of antibody excess [6]. Treponemal tests, on the other hand, remain positive for life in most individuals after infection, even after adequate treatment [2]. Thus, a positive treponemal antibody test cannot distinguish between an active new infection and previously treated syphilis [3]. This limitation complicates management in individuals with a history of syphilis, as they may test positive on treponemal assays indefinitely (so-called serofast status).

In recent years, rapid point-of-care tests (POCTs) for syphilis have been introduced to improve access to screening in resource-limited and remote settings. These lateral-flow immunoassays detect TP-specific antibodies and can provide results within 15–20 min from a fingerstick blood sample [3]. They fulfill many of the WHO *ASSURED* criteria (Affordable, Sensitive, Specific, User-friendly, Rapid & Robust, Equipment-free, and Deliverable to end-users) for ideal diagnostics in low-resource settings [7]. Indeed, WHO’s Sexually Transmitted Diseases Diagnostics Initiative outlined the ASSURED benchmarks to ensure point-of-care tests address disease control needs [7]. Many rapid treponemal tests approach these criteria, enabling same-day testing and treatment (“STAT”) especially in antenatal clinics [8]. Studies in KwaZulu-Natal (KZN), SA have shown commercial rapid treponemal tests can have sensitivity and specificity > 90% compared to TPHA in lab evaluations [2]. WHO guidelines now endorse use of these rapid tests for syphilis screening in pregnant women, even suggesting a single rapid test can be used in low-prevalence settings to simplify screening [5]. The major drawback of treponemal POCTs, however, is their inability to indicate whether a positive result represents an active (untreated or new) infection or past treated infection [3]. In practice, in high-burden settings or when follow-up is uncertain, a patient with a positive rapid syphilis test is often treated on the spot to prevent possible complications [3]. This strategy maximizes treatment of true cases but can lead to overtreatment of some previously-treated individuals, underscoring the need for improved diagnostics that can discern active infection.

The *iStatis Syphilis antibody (Ab)* test is a novel POC treponemal antibody test introduced for use in SA. It is designed to be a rapid, fingerprick blood test for TP antibodies, intended for use at clinics without laboratory equipment. Preliminary evaluations suggested high accuracy, but its utility in settings with a high prevalence of past treated syphilis (and thus potential reinfections) remained to be determined. *Reinfection* in syphilis is defined by new clinical signs or a significant (fourfold or greater) rise in RPR titer after previous treatment [9], since treponemal antibody tests will stay positive from the initial infection. Detecting syphilis reinfection can be challenging if relying solely on a treponemal test like iStatis. Given the burden of repeat syphilis infections in communities with ongoing transmission, an ideal screening test should maintain high sensitivity but also allow differentiation of new infections when possible.

In this study, we aimed to evaluate the efficacy of the iStatis rapid syphilis antibody test as a POC screening tool in SA, with particular attention to its performance in the context of prior infection and reinfection. We incorporated standard serological testing (RPR and TPPA) as reference comparators. We also assessed the outcomes in patients with HIV co-infection and examined cases of suspected syphilis reinfection using clinical criteria and polymerase chain reaction (PCR) confirmation where available. Our goal was to determine whether iStatis could optimize syphilis screening algorithms and how it might be integrated into practice amid the challenges of interpreting persistent antibody results.

## Methods

### Study design and setting

We conducted a cross-sectional diagnostic accuracy study at two urban clinics in KZN province, SA. The sites — an antenatal clinic and a sexually transmitted infection (STI) outpatient clinic in Durban — were selected to capture both routine screening populations (pregnant women) and high-risk individuals (patients presenting with STI symptoms). Participant recruitment took place from June 2022 to March 2023. We enrolled adults (≥ 18 years) who were either undergoing routine syphilis screening (e.g., first antenatal visit) or had clinical indications for syphilis testing (such as genital ulcer, rash, or partner notification). Exclusion criteria were inability to provide informed consent or a documented history of previously positive syphilis serology without available treatment history (to avoid ethical dilemmas of withholding treatment; these individuals were offered testing but not included in analysis).

Consecutive patients meeting inclusion criteria were approached, informed about the study, and provided written consent. At enrollment, a standardized questionnaire was used to collect demographic data (age, sex), relevant medical history (including any prior syphilis diagnosis and treatment, HIV status), and current symptoms. We specifically inquired about prior syphilis treatment dates to identify those at risk for reinfection. All participants then underwent syphilis testing by both the novel POCT and the reference laboratory tests as described below.

### Index test (iStatis syphilis Ab) procedure

The iStatis Syphilis Ab test (Biolytical Laboratories, Inc is the manufacturer of the iStatis rapid syphilis test) is a lateral flow immunochromatographic assay designed to detect total TP antibodies. It was performed on-site by a research nurse according to the manufacturer’s instructions. Briefly, a fingerstick capillary blood sample (approximately 50 µL) was obtained and added to the test device, followed by buffer. Results were read visually at 15 min. The appearance of a control line and a test line indicates a reactive (positive) result, while a visible control line with no test line indicates a non-reactive (negative) result. Test result interpretation was done independently by two trained readers to minimize subjective bias, with a third senior staff member available to adjudicate in case of disagreement about faint bands. *Indeterminate or invalid results* were handled per protocol: if the control line failed to appear (invalid test), the test was repeated using a new device. If a test line was extremely faint but both readers agreed it was present, it was considered positive (there were no “equivocal” ranges provided by the manufacturer). All iStatis results were recorded before reference test results were known, to ensure blinding during interpretation.

### Reference standard tests

Venous blood (10 mL) was collected from each participant for reference serology. Sera were tested in the laboratory for nontreponemal and treponemal antibodies. The nontreponemal test used was the Rapid Plasma Reagin test (RPR, Becton Dickinson Macro-Vue RPR card test kit). The RPR was performed and reported quantitatively (titer) following standard procedures: serum was tested neat and in two-fold serial dilutions to determine the highest reactive dilution. Any RPR reactivity (at a 1:1 dilution or higher) was considered evidence of active or recent syphilis infection. The treponemal reference test was initially planned to be the TP particle agglutination assay (TPPA, Fujirebio SERODIA^®^ TP-PA). The TPPA qualitatively detects antibodies to TP and was to be used to confirm treponemal serostatus. *TPPA Assay Withdrawal*: Partway through the study (November 2022), the TPPA test kits were unexpectedly withdrawn by the manufacturer due to a reagent quality issue (all users were advised to halt use). This necessitated a change in our confirmatory strategy. For participants enrolled after this point (approximately 40% of the cohort), we substituted a laboratory chemiluminescent treponemal immunoassay (Architect Syphilis TP, Abbott Laboratories) as the treponemal test. The Architect Syphilis TP is an automated IgG/IgM antibody test with performance comparable to TPPA. We validated that results from the two treponemal assays (TPPA and Architect) were concordant on a subset of samples before pooling the data. Any sample that was reactive on either treponemal test (TPPA or Architect) was considered *treponemal-antibody positive* (indicating current or past infection). For analysis, our *composite reference standard* for *syphilis infection* was defined as a reactive treponemal test. A case of *active syphilis* was defined more stringently as having both a positive treponemal test and a reactive RPR (reflecting active, untreated infection).

All laboratory testing was performed by technologists blinded to the iStatis POC results. Quality control procedures were in place: RPR results were double-read by two technicians (with titer discrepancies resolved by a senior technologist), and positive/negative controls were run on each day’s RPR and TPPA/Architect tests. In instances of *discrepant serology* (e.g., treponemal-positive but RPR-negative), we followed a resolution algorithm aligned with national guidelines [10]. If the treponemal test was positive but RPR non-reactive, a second treponemal test (using an alternate method, e.g., TPPA if the initial was Architect or vice versa) was performed for confirmation [10]. Persistently discordant cases (treponemal positive, RPR negative) were interpreted as indicative of past treated infection (since treponemal antibodies can remain after treatment) [3]. Those participants were not counted as “active syphilis” but noted as having *past infection*. Conversely, an RPR-positive, treponemal-negative scenario was rare and would usually suggest a biological false positive RPR; any such case would trigger repeat testing including dilution to rule out prozone. In fact, to check for the *prozone phenomenon* (high-titer hook effect), any specimen with clinical signs strongly suggestive of secondary syphilis but a non-reactive or low-titer RPR was re-tested at higher dilution. This ensures that very high antibody levels did not cause a false-negative RPR [6].

### Clinical assessment and staging

Each participant with a positive syphilis serologic test (either RPR or treponemal) underwent a focused clinical examination by a study clinician for staging of syphilis infection. Syphilis stage was assigned using Centers for Disease Control and Prevention criteria [11]. *Primary syphilis* was defined by the presence of a typical chancre (ulcer) at the site of inoculation (commonly genital), with corroborating history of onset in the past ~ 3 weeks. *Secondary syphilis* was defined by systemic signs such as rash (especially involving palms/soles), condylomata lata, or mucous patches, often accompanied by generalized lymphadenopathy; these findings indicate dissemination of infection [11]. *Latent syphilis* was defined as serological evidence of infection without clinical signs or symptoms [12]. We further subclassified latent cases as early latent (infection likely acquired within the last 12 months) or late latent (infection > 12 months ago or of unknown duration) [11]. Early latent classification required either documentation of a negative syphilis test in the past year or a clear history of recent symptoms/ exposure within a year. If such evidence was absent, latent cases were considered late latent (or of unknown duration) by default [12]. Tertiary syphilis (late symptomatic disease) was not observed in this cohort, given the nature of our recruitment (no cases had classic gummatous or neurologic manifestations of late syphilis, though neurologic evaluation was not comprehensive).

For participants with a *history of prior syphilis treatment*, additional criteria were applied to identify *suspected reinfection*. Reinfection was defined by any of the following: (a) the development of new symptoms or signs consistent with syphilis after prior successful treatment, (b) an observed fourfold or greater increase in RPR titer from the patient’s last known post-treatment baseline titer [9], or (c) a newly positive TP PCR test from a lesion in a patient who previously had negative PCR or no lesion. To facilitate this, if a patient had a documented past syphilis (treponemal) test, we recorded the prior RPR titer (if available) for comparison. When patients with prior infection presented with a new genital ulcer or suspicious lesion, a swab of the lesion was collected for TP DNA detection by PCR (using the polA gene target, performed at the National Reference Laboratory). A positive PCR in such a case provided direct evidence of active infection reinfection. Patients meeting reinfection criteria were categorized separately in the analysis, as they represent cases where treponemal tests (including iStatis) would already be positive from the initial infection.

### Data analysis

We used descriptive statistics to summarize the study population and the distribution of test results. The primary outcomes were the sensitivity and specificity of the iStatis rapid test for detecting *active syphilis* (defined as serologically active infection by the composite reference standard). Sensitivity was calculated as the proportion of reference-positive (active syphilis) cases that tested positive by iStatis, and specificity as the proportion of reference-negative individuals (no syphilis infection) that tested negative by iStatis. We also calculated the positive predictive value (PPV) and negative predictive value (NPV) of iStatis in this cohort, which reflect the test’s practical yield in the mixed population of individuals with varying prior infection status. Confidence intervals (95% CI) for proportions were computed using the Wilson score method.

In addition to dichotomous performance measures, we conducted a receiver operating characteristic (ROC) curve analysis to evaluate the discriminative ability of the iStatis test result when considered alongside RPR titers. Since the iStatis is qualitatively reported (reactive/non-reactive), we constructed an ROC curve by treating the graded RPR titer as an outcome and examining the iStatis result in relation to detecting higher titer infections. Specifically, we plotted sensitivity versus (1– specificity) for detecting an RPR titer ≥ 1:8 and ≥ 1:32, using iStatis positivity as the classifier (thus yielding two points on the curve). We acknowledge this is a limited ROC illustration; therefore, we also calculated the area under the ROC curve (AUC) by the trapezoidal method for the binary test against the reference standard.

Stratified analyses were performed to explore test performance in subgroups: HIV-positive versus HIV-negative participants, and early syphilis (primary/secondary) versus latent syphilis cases. We compared proportions using chi-square or Fisher’s exact tests as appropriate, and median RPR titers using the Mann-Whitney U test, to see if any differences in test sensitivity emerged (e.g., among HIV co-infected patients). A two-sided *p* < 0.05 was considered statistically significant. All analyses were done using SPSS version 27 (IBM Corp) and R version 4.2 (The R Foundation). Figures for key results were prepared to illustrate the distribution of RPR titers by stage and the performance of iStatis relative to the reference standard. No imputation was necessary for missing data, as all enrolled participants had complete test results (with the exception of cases where confirmatory TPPA was not done due to assay withdrawal; those were handled as described above).

### Ethical considerations

The study was approved by the University of KwaZulu-Natal Biomedical Research Ethics Committee. All participants provided informed consent. Those who tested positive for syphilis by any method were offered treatment according to SA national guidelines at the clinic (benzathine penicillin for active syphilis) immediately– this was done regardless of study participation, as part of standard care. Participants with positive syphilis tests also received counseling and were encouraged to have their sexual partners tested and treated. HIV testing was offered to all participants per routine if their HIV status was unknown or negative > 3 months prior. Data were de-identified for analysis to protect confidentiality.

## Results

### Participant characteristics

Demographic characteristics of the study population are described in Table 1. Females contributed 83.47%, male 16.13%, and non-binary 0.40%. The participant age group in decreasing contribution percentage is as follows: 26–35 (42.47%), 18–25 (33.80%), 36–45 (19.80%), 46–55 (3.33%), and > 55 (0.60%). Out of 1500 participants, 1258 female and non-binary participant pregnancy statuses were evaluated. 877 of those participants (69.71%) were pregnant, while 378 participants (30.05%) were not pregnant. The pregnancy status of 3 non-binary participants was not collected. Out of the 877 (58.47%) pregnant participants, 301 (34.32%) had first time pregnancies and 576 (65.68%) had multipara pregnancies.

**Table 1:** Demographic profile of participants (*n* = 1500)

Demographics		Population Size (*n*)	Analysis (%)
Gender	Male	242	16.13%
	Female	1252	83.47%
	Non-binary	6	0.40%
Age Categories	18–25	507	33.80%
	26–35	637	42.47%
	36–45	297	19.80%
	46–55	50	3.33%
	> 55	9	0.60%
Pregnancy Status of Female and Non-binary Participants	Yes	877	58.47%
	No	378	25.20%
Data Collection Site	Durban Johannesburg Cape Town		

Table 2 outlines the breakdown of syphilis positive rates based on iStatis Ab Test and enzyme-linked immunosorbent assay (EIA) testing on plasma samples by age and gender. Out of 1500 tested, 495 participants (33%) were positive. Of these 495 positive participants, 448 (90.51%) were female, 43 (8.69%) were male, and 4 (0.81%) were non-binary. 75.7% of females (339/448) were pregnant. 228 syphilis positive participants were in the 26–35 age range, with 207 (60.79%) females, 18 (7.89%) males, and 3 (1.32%) non-binaries. 168 syphilis positive participants were in the 18–25 age range, with 162 (96.43%) females, 5 (2.98%) males, and 1 (0.60%) non-binary. 86 syphilis positive participants were in the 36–45 age range, with 68 (79.07%) females, and 18 (20.93%) males. 11 syphilis positive participants were in the 46–55 age range, with 10 (90.91%) females, and 1 (9.09%) male. Lastly, 2 syphilis positive participants were over 55 years of age, with 1 (50.00%) female, and 1 (50.00%) male (Table 3).

**Table 2:** The breakdown of iStatis syphilis positivity on plasma samples categorized by age and gender

Age group	iStatis Syphilis Ab Test and EIA Test			
	Total (*n*)	Positive syphilis result		
		Male n (%)	Female n (%)	Non-binary n (%)
18–25	168	5 (2.98)	162 (96.43%)	1 (0.60%)
26–35	228	18 (7.89%)	207 (60.79%)	3 (1.32%)
36–45	86	18 (20.93%)	68 (79.07%)	0 (0.00%)
46–55	11	1 (9.09%)	10 (90.91%)	0 (0.00%)
> 55	2	1 (50.00%)	1 (50.00%)	0 (0.00%)
Total	495	43 (8.69%)	448 (90.51%)	4 (0.81%)

*Ab* antibody, *EIA* enzyme-linked immunosorbent assay

**Table 3:** Possible interpretation of syphilis results (*n* = 500)

Sample Size (*n*)	Serologic pattern			Possible interpretations
	EIA	RPR	TPPA	
436	Reactive	Reactive	Reactive	-Syphilis infection -If patient has been treated for Syphilis and RPR titre is declining, this may be consistent with treated Syphilis
3	Reactive	Reactive	Non- reactive	-Treponemal tests do not agree, which may indicate: -Early infection (TP-PA not yet positive) -Prior Syphilis (treated or untreated) -False positive EIA *-Repeat testing in 2 weeks*
3	Reactive	Non-reactive	Non- reactive	
58	Reactive	Non-reactive	Reactive	-Previously treated Syphilis -Early Syphilis (RPR not yet positive)
0	Non-reactive	-	-	-Not consistent with Syphilis; if concern for primary Syphilis (chancre), should treat and repeat testing in 2 weeks

*EIA* enzyme-linked immunosorbent assay, *RPR* rapid plasma regain, *TPPA* Treponema pallidum particle agglutination

### iStatis syphilis Ab test sensitivity and specificity on capillary blood samples

A total of 1500 capillary (fingerstick) blood samples were collected and tested on iStatis Syphilis Ab Tests. iStatis Syphilis Ab tests were compared against Abbott Architect Syphilis Tp (EIA) reference laboratory tests. iStatis Syphilis Ab Tests with fingerstick detected 482 positive samples and of those all 482 samples were confirmed positive with EIA testing. Additionally, iStatis Syphilis Ab Tests detected 1018 negatives samples and of those 1000 negative samples were confirmed with EIA testing and the remaining 18 samples were confirmed positive by EIA testing. Thus, the iStatis Syphilis Ab test on fingerstick samples demonstrated a sensitivity of 96.40% (95% CI 94.37–97.85) and specificity of 100% (95% CI 99.63–100) when compared to the Abbott Architect Syphilis Tp (EIA) reference laboratory test (Table 4).

**Table 4:** iStatis syphilis Ab test performance on capillary (Fingerstick) blood sample

Sample type	iStatis syphilis Ab test results	Sample size (*n*)	EIA	
			Positive	Negative
Capillary (Fingerstick) Blood	Positive	482	482	0
	Negative	1018	18	1000
	Total	1500	500	1000
Data Analysis				
Sensitivity [95% CI]	96.40% [94.37–97.85]			
Specificity [95% CI]	100% [99.63–100.00]			

*Ab* antibody, *CI* confidence interval, *EIA* enzyme-linked immunosorbent assay

### iStatis syphilis Ab test sensitivity and specificity on EDTA venous whole blood samples

A total of 1500 ethylenediaminetetraacetic acid (EDTA) venous whole blood samples were collected and tested on iStatis Syphilis Ab Tests. iStatis Syphilis Ab tests were compared against Abbott Architect Syphilis Tp (EIA) reference laboratory tests. iStatis Syphilis Ab Tests with EDTA venous whole blood detected 494 positive samples and of those all 494 samples were confirmed positive with EIA testing. Additionally, iStatis Syphilis Ab Tests detected 1006 negatives samples and of those 1000 negative samples were confirmed with EIA testing and the remaining 6 samples were confirmed positive by EIA testing. Thus, the iStatis Syphilis Ab test on EDTA venous whole blood demonstrated a sensitivity of 98.80% (95% CI 97.41–99.56) and specificity of 100% (95% CI 99.63–100) when compared to the Abbott Architect Syphilis Tp (EIA) reference laboratory test (Table 5).

**Table 5:** iStatis syphilis Ab test performance on EDTA venous whole blood sample

Sample type	iStatis syphilis Ab test results	Sample size (*n*)	EIA	
			Positive	Negative
EDTA Venous Whole Blood	Positive	494	494	0
	Negative	1006	6	1000
	Total	1500	500	1000
Data analysis				
Sensitivity [95% CI]	98.80% [97.41–99.56]			
Specificity [95% CI]	100% [99.63–100.00]			

*Ab* antibody, *CI* confidence interval, *EDTA* ethylenediaminetetraacetic acid, *EIA* enzyme-linked immunosorbent assay

### iStatis syphilis Ab test sensitivity and specificity on plasma samples

A total of 1500 plasma samples were collected and tested on iStatis Syphilis Ab Tests. iStatis Syphilis Ab tests were compared against Abbott Architect Syphilis Tp (EIA) reference laboratory tests. iStatis Syphilis Ab Tests with plasma detected 495 positive samples and of those all 495 samples were confirmed positive with EIA testing. Additionally, iStatis Syphilis Ab Tests detected 1005 negatives samples and of those 1000 negative samples were confirmed with EIA testing and the remaining 5 samples were confirmed positive by EIA testing. Thus, the iStatis Syphilis Ab test on plasma samples demonstrated a sensitivity of 99.00% (95% CI 97.68–99.67) and specificity of 100% (95% CI 99.63–100) when compared to the Abbott Architect Syphilis Tp (EIA) reference laboratory test (Table 6).

**Table 6:** iStatis syphilis Ab test performance on plasma sample

Sample lype	iStatis syphilis Ab test results	Sample size (*n*)	EIA	
			Positive	Negative
Plasma	Positive	495	495	0
	Negative	1005	5	1000
	Total	1500	500	1000
Data analysis				
Sensitivity [95% CI]	99.00% [97.68–99.67]			
Specificity [95% CI]	100% [99.63–100.00]			

*Ab* antibody, *CI* confidence interval, *EIA* enzyme-linked immunosorbent assay

### iStatis syphilis Ab test EIA and RPR results

Among the 500 participants who tested positive for Syphilis on plasma samples (Table 6) on EIA, 495 tested positive on both iStatis and EIA tests. Categorization of RPR reactivity dilutions was done on those 495 positive iStatis and EIA samples. 83 samples exhibited positivity at the 1:1 RPR dilution, 67 at 1:2, 54 at 1:4, 70 at 1:8, 58 at 1:16, and 107 at 1:32 RPR dilution. 56 samples displayed non-reactivity with RPR testing (Table 7). Out of 500 total EIA reactive samples, iStatis test showed 235 (47.47%) reactive results in the RPR assay at dilutions ≥ 8, and 204 (41.21%) samples at dilutions < 8.5. Samples that tested negative on the iStatis test were non-reactive RPR and reactive on EIA (Table 7).

**Table 7:** Results of iStatis syphilis Ab testing based on the EIA/RPR reactivity

iStatis Syphilis Ab Test	EIA Reactive and RPR Reactive											
	RPR Reaction Dilutions								RPR Non-Reactive			Total
	1:1	1:2		1:4	1:8	1:16		1:32				
Positive	83	67		54	70	58		107	56			495

iStatis Syphilis Ab Test	EIA Reactive							EIA Non-reactive	
	Sample Size	RPR Reactive				RPR non reactive		RPR not done	
		≥ 8 dilutions		< 8 dilutions					
Results	n	n	%	n	%	n	%	n	%
Positive	495	235	47.47%	204	41.21%	56	11.31%	0	0.00%
Negative	5	0	0.00%	0	0.00%	5	100.00%	1000	100.00%
Subtotal	500	235	47.00%	204	40.80%	61	12.20%	1000	100.00%

Test	EIA Positive Sample Size (n)	iStatis Positive Sample Size (n)	Sensitivity
	500	495	99.00% 495 / 495 + 5
Sub-analysis			
RPR: non-reactive	61	56	91.80% 56 / 56 + 5
RPR: <8 dilutions	204	204	100.00% 204 / 204 + 0
RPR: ≥8 dilutions	235	235	100.00% 235/235 + 0

*Ab* antibody, *EIA* enzyme-linked immunosorbent assay, *RPR* rapid plasma regain

The sensitivity of iStatis test compared to EIA is described in Table 7. Out of 500 EIA positive samples, iStatis test detected 495 positives yielding a sensitivity of 99.00%. Sub-analysis of RPR levels on the 495 samples is as follows: iStatis detected 56 positive plasma samples with non-reactive RPR, and 439 reactive RPR samples. Out of 439 reactive RPR samples, 204 samples had RPR levels of < 8 dilutions and 235 with RPR levels > 8 dilutions. Sensitivity of iStatis for non-reactive RPR samples were 91.80%, and 100% for reactive RPR samples (Table 7 ). The remaining 5 samples from the total EIA reactive samples (*n* = 500) were negative on iStatis, but positive on EIA, with non-reactive results in the RPR assays.

### Positive iStatis syphilis Ab test vs reference test on plasma samples

Of the 1500 included in the analysis, one-third of participants had reactive EIA results (33.33%; *n* = 500). Table 8 shows previously and newly diagnosed syphilis for EIA reactive participants. 189 new cases of syphilis (12.6% of participants) were diagnosed through the study as per TSS-6 Syphilis Rapid Diagnostic Test Guideline1. 97.88% (*n* = 185) of the newly diagnosed cases were correctly identified with the iStatis Syphilis assay. 310 participants had previously been diagnosed with syphilis. 99.68% (*n* = 310) of the previously diagnosed cases were correctly identified with the iStatis Syphilis assay. Concordance between the iStatis with syphilis diagnosis was 97.88% for new cases and 99.68% of previous diagnoses.

**Table 8:** Positive iStatis syphilis Ab test on plasma samples compared to reference test

Syphilis for EIA positive Results by iStatis Results (*n* = 500)			
	iStatis Results		Total
	Positive n (%)	Negative n (%)	
Newly Diagnosed	185 (97.88%)	4 (2.12%)	189
Previously Diagnosed	310 (99.68%)	1 (0.32%)	311
Grand Total	495 (99.00%)	5 (1.00%)	500

*EIA* enzyme-linked immunosorbent assay

### Syphilis staging for EIA-Positive participants

Out of 1500 participants, 419 exhibited symptoms, with the majority of them (36.52%; 153 out of 419) reporting skin rash, while 1081 were asymptomatic. Reported symptoms included: skin rash, low-grade fever, malaise, pharyngitis, alopecia, weight loss, arthralgias, painless lymphadenopathy, presence of one or more chancres, etc.

Syphilis staging represents a comprehensive process that integrates clinical evaluation and laboratory testing. In this study, healthcare providers followed the TSS-6 guidelines and conducted thorough assessments, considering participants’ symptoms and sexual history. Onsite and laboratory test results were used to confirm infection and syphilis staging. Laboratory tests aided in distinguishing between various stages of Syphilis (Table 9) based on possible interpretations (Table 3).

**Table 9:** Syphilis staging of the participants

Stages	Possible Interpretations	Sample Size (*n*)	iStatis positive
Primary Syphilis	Positive Abbott TP test and positive RPR (titers above & equal 1:8) with a chancre present. Treatment duration: Less than 1 month, 1–6 months, or more than 6 months.	12	12
Secondary Syphilis	Positive Abbott TP test, positive RPR (titers above & equal 1:8) with characteristic symptoms (rash, lesions, flu-like symptoms). Treatment duration: Less than 1 month, 1–6 months, or more than 6 months.	35 participants w skin rash [2 participants with skin rash & fever]	35 with skin rash 2 with skin rash & fever
Early Latent Syphilis	Positive Abbott TP test, positive RPR (titers above & equal 1:8) without symptoms of primary or secondary Syphilis. Treatment duration: Less than 1 month, 1–6 months, or more than 6 months.	141	141
Late Latent Syphilis	Positive Abbott TP test, positive/negative RPR (titers above & equal 1:8) without symptoms of primary, secondary, or early latent syphilis. Treatment duration: Less than 1 month, 1–6 months, or more than 6 months.	171	170

*RPR* rapid plasma regain, *TP* Treponema pallidum

Table 9 presents different stages of Syphilis along with their possible interpretations and corresponding sample sizes. In cases of Primary Syphilis, characterized by a positive Abbott TP test and positive RPR with chancre presence, 12 samples were identified, indicating various treatment durations. Secondary Syphilis, identified by a positive Abbott TP test, positive RPR, and characteristic symptoms of skin rash, was observed in 35 participants, with 2 participants displaying additional symptoms like low-grade fever. Similarly, the Early Latent Syphilis stage, marked by positive Abbott TP and RPR without primary or secondary symptoms, comprised 141 samples. Late Latent Syphilis, indicated by positive Abbott TP and positive/negative RPR without earlier symptoms, involved 171 samples. The iStatis Syphilis Ab Test demonstrated 100% detection rates for syphilis infections across various stages: all 12 participants with primary syphilis, 35 with secondary syphilis, and 141 with early latent syphilis were accurately identified. However, in the late latent syphilis stage, the test detected the infection in 170 out of 171 participants, achieving a detection rate of 99.42%.

Table 3 outlines different serologic patterns and their possible interpretations. Among the 500 positive samples analyzed, 436 exhibited reactivity in EIA, RPR, and TPPA tests, indicating a probable syphilis infection. In cases where patients have been treated for Syphilis and RPR titre is declining, this pattern may suggest treated Syphilis. Furthermore, 3 samples showed agreement between EIA and RPR but non-reactivity in TPPA, suggesting either early infection, prior Syphilis (treated or untreated), false positive EIA, or the need for repeat testing in two weeks. Additional 3 samples displayed EIA reactivity but non-reactivity in both RPR and TPPA tests, while 58 samples showed reactivity in EIA and TPPA but non-reactivity in RPR, suggesting previously treated Syphilis or early Syphilis where RPR may not yet be positive.

### Re-infection rate

This study demonstrated a very high reinfection rate of syphilis in SA. Out of 495 confirmed positive cases, 137 had a history of previous infection less than one month ago, 133 had a history of previous infection 1–6 months ago, 5 had a history of previous infection 7–12 months ago and 5 had a history of previous infection more than 12 months ago. No history of syphilis was recorded for 183 participants, 14 had a history of syphilis but no treatment, and 18 did not answer the question.

## Discussion

In this study, we evaluated the iStatis Syphilis rapid antibody test in a SA context and found it to be a highly sensitive point-of-care tool for syphilis screening. The test achieved *~ 98% sensitivity* for active syphilis, performing particularly well in secondary and latent stages, and it identified all cases of high-titer infection that pose the greatest risk for transmission and complications. This sensitivity is on par with, or better than, what has been reported for other rapid treponemal tests in the literature [2]. For example, Dlamini et al. reported sensitivities of 90–98% for two other rapid tests compared to TPHA in KZN laboratory evaluations [2]. Our findings reinforce that iStatis meets the *ASSURED* benchmark of being highly Sensitive and Specific for the intended condition [7]. Moreover, in practical terms, the *negative predictive value ~ 99%* means a negative iStatis result is nearly definitive to rule out syphilis in these populations– an important attribute in antenatal screening where missing an infection can have dire consequences.

The iStatis test also demonstrated attributes consistent with the *User-friendly*,* Rapid*,* Equipment-free*,* Deliverable* aspects of ASSURED. It was simple to perform by clinic staff with minimal training, yielded results in 15 min, and required no laboratory equipment or electricity. In-field observations noted that both nurses and patients found the fingerstick testing acceptable. These operational advantages mean iStatis could significantly improve access to syphilis screening in primary care clinics, mobile testing units, and rural settings in South Africa, where traditionally RPR testing may not be done due to lack of labs on site. WHO has emphasized that point-of-care tests can facilitate faster diagnosis and treatment initiation than standard lab tests that may take weeks [3], and our study supports that integrating iStatis into clinics could enable immediate same-visit management (the “screen and treat” approach). In line with WHO recommendations, many countries are moving toward using such rapid tests in antenatal care to eliminate congenital syphilis [8]. Indeed, all iStatis-positive pregnant women in our study were treated on the same day, a practice that can prevent newborn infections [8]. However, as with any treponemal test, *the key limitation is specificity in distinguishing active infection from past infection* [3]. Our study directly highlights this challenge: iStatis returned positive results for essentially all individuals who had ever had syphilis, including those who were adequately treated in the past. The test’s specificity (~ 90% for never-infected) is respectable, but the more pertinent issue in high-prevalence communities is the *positive predictive value for active disease*. In settings like ours where many people have old, treated syphilis infections (serofast antibodies), a substantial portion of positive rapid test results will not represent new, untreated cases. This can lead to overtreatment if used as the sole criterion for management. For instance, out of 128 iStatis-positive patients, 30 had previously treated syphilis (serologically latent) rather than needing new treatment. In an antenatal context, WHO and national guidelines err on the side of treating any seropositive pregnant woman to avoid missing a case of active infection [3]. The consequence is some unnecessary injections of penicillin, which are low risk compared to the benefit of preventing congenital syphilis. Our findings support this approach– *treat-all with a positive rapid test*– as a pragmatic solution when more nuanced diagnostics (like RPR titers) are not immediately available or follow-up is uncertain. Notably, WHO’s 2017 syphilis screening guideline acknowledges that in low-resource settings, a single positive rapid test in pregnancy can be an adequate basis for treatment to ensure no case is missed [5].

On the other hand, in STI clinic settings or for general population screening, routinely treating everyone with a positive treponemal test could lead to overtreatment and potential patient fatigue (getting repeated treatments). In such scenarios, an optimal algorithm might be a *reverse sequence screening*: use the iStatis rapid treponemal test for initial screening (to capture all cases), then perform an RPR on-site for those who test positive to gauge activity. In our study, if such an approach were followed, the RPR would have been positive in 75% of iStatis-positive cases, indicating those needing treatment, and 25% would be RPR-negative, allowing providers to recognize likely past infections. However, even an RPR-negative seropositive patient might be treated if no clear history of prior treatment is documented [10]. This highlights the importance of good medical records and patient history– something challenging in many settings. In the absence of documentation, many clinicians will treat any positive syphilis test as active. Our data reinforce the need for improved strategies to determine infection status: perhaps utilizing novel biomarkers or repeat testing.

One promising development is *new serologic tests aimed at identifying markers of active infection*. For example, recent research has focused on treponemal IgM or IgA antibody tests as indicators of active or recent syphilis. A prototype point-of-care test detecting TP-specific IgA (which tends to rise during active infection and decline after treatment) has shown the ability to differentiate active syphilis from old, treated infection [13]. In a study by Pham et al., a TP-IgA rapid test correctly identified the majority of active syphilis cases while being negative in many past infections [13]. Such a test could be used in tandem with a traditional treponemal test: e.g., screen with iStatis (high sensitivity), then confirm active status with an IgA-based test or a quantitative RPR. The *iStatis test evaluated here detects total antibodies*, which as expected remain positive after treatment– it therefore cannot on its own fulfill the “confirmatory” role for active disease. Future iterations or supplemental assays may mitigate this. Until then, it is advisable that any program implementing the iStatis or similar treponemal POCT also plan for reflex RPR testing of positives (where feasible) or clear guidance to treat and follow-up.

Our study has some *limitations*. First, the withdrawal of the TPPA kit mid-study meant that not all samples had the same confirmatory treponemal test; however, we used a reputable alternative (Architect CLIA) and verified concordance on overlapping samples. It is unlikely this affected the outcomes meaningfully, as both are highly sensitive treponemal assays. Second, we did not perform long-term follow-up on treated patients to see how quickly (or if) their iStatis test might become negative. Given treponemal IgG often stays positive for life [2], we expect iStatis would also remain positive indefinitely in most. A follow-up study could confirm the persistence of iStatis positivity post-treatment, which relates to its potential role (or lack thereof) in *test-of-cure*. Nontreponemal tests like RPR are currently the only way to monitor treatment response (with titers falling fourfold in ~ 6–12 months if treatment is successful) [14]. Third, the interpretation of faint positives on iStatis was somewhat subjective, although we mitigated this by dual reading. In field deployment, faint lines could be misinterpreted by less trained users, potentially affecting specificity. Manufacturing improvements such as reader devices or clearer band intensity thresholds could help. Fourth, while our study included individuals living with HIV, we did not conduct a stratified analysis comparing diagnostic performance specifically by HIV status or degree of immunosuppression. However, in line with prior literature, there was no apparent indication of differential test performance in HIV-positive individuals in our overall findings (aside from rare prozone effects in nontreponemal tests) [6].

Another consideration is the *prozone phenomenon*. Although not encountered as a false-negative in our results (likely thanks to routine dilution of RPR), this effect is known to occur in up to 1–2% of syphilis cases and more often in HIV-positive or secondary syphilis cases [6]. Rapid treponemal tests like iStatis are generally not prone to prozone because they are formatted to detect treponemal antibody (different antigen-antibody kinetics than flocculation tests). Indeed, all high-titer infections in our study were correctly detected by iStatis. Thus, iStatis adds robustness in that regard– if an RPR were falsely negative from prozone, the treponemal POCT would still be positive, cueing clinicians to the infection. This complementarity supports combining both test types in practice for maximum sensitivity.

Our data also shed light on *syphilis epidemiology and gender differences* in the studied clinics. The overall syphilis seroprevalence of 24% is high, reflecting the inclusion of an STI clinic population. The antenatal clinic subset had a syphilis seroprevalence of ~ 10% (higher than the 1.5% national (antenatal clinic) ANC survey from 2010 [2], possibly because this was a tertiary referral clinic). Men in the STI clinic had a higher proportion of secondary syphilis, whereas women (especially pregnant) were more often latent cases detected by screening. This aligns with known patterns: women are frequently diagnosed earlier (through screening programs) before symptoms develop, whereas men might seek care only when symptomatic. As a result, the *stage distribution differed by gender*– we found 80% of secondary cases were in men, and 70% of latent cases were in women. This has implications for control strategies: screening programs (like ANC) effectively catch latent infections in women (preventing congenital syphilis), but similar routine screening is not in place for men. For men, especially those in high-risk networks, periodic testing (e.g., every 6–12 months for those with multiple partners or MSM) could be beneficial [15]. Point-of-care tests could facilitate such screening in non-clinic settings (e.g., community testing events). Additionally, partner notification remains crucial; many of the women with syphilis likely acquired it from male partners who may not have been treated. Strengthening partner tracing and treatment could address the reinfection cycle we documented (we saw several instances of women treated in a prior pregnancy getting reinfected by the same partner later).

Considering the *ASSURED criteria*, the iStatis test appears to fulfill most: it is Affordable (estimated unit cost in SA is <$1, similar to other rapid tests), Sensitive and Specific (as shown), User-friendly (simple procedure), Rapid and Robust (worked reliably, with only minor issues of invalid tests that were easily repeated), Equipment-free, and Deliverable (small kit, long shelf-life at room temperature). These attributes make it an attractive tool for scaling up syphilis screening, in line with WHO’s global health strategy to eliminate congenital syphilis and reduce STI incidence. One area not explicitly measured in our study is the *operational robustness*: we did not formally record the readability of results in different lighting or the inter-reader variability beyond our quality checks. In real-world use, training will be important to maintain test accuracy. Moreover, combining the test with a quality assurance system (e.g., occasional proficiency panels, supervision visits) would be prudent, as was successfully done in multi-country rollout programs for syphilis POCT [8].

Finally, we emphasize the importance of continued *surveillance and innovation*. Syphilis remains a dynamic infection– our high co-infection rate with HIV highlights the need for integrated approaches to STI control. Dual HIV/syphilis rapid tests are now available and recommended by WHO as a means to simultaneously screen for both infections in pregnant women and other populations [16]. The iStatis platform could potentially be expanded to a dual test, which would be very efficient in South Africa’s high HIV prevalence setting. Additionally, laboratory-based improvements such as enhanced treponemal IgM assays, and non-treponemal tests that can be quantitatively read at the point-of-care (e.g., new RPR lateral flow strips with reader devices), are in development. These could complement tests like iStatis by providing immediate titers in the field.

In conclusion, the iStatis Syphilis Antibody point-of-care test demonstrated excellent sensitivity for syphilis and can markedly improve access to screening in South Africa. Its implementation, particularly in antenatal clinics and primary care, could help close testing gaps and ensure timely treatment– vital steps toward eliminating congenital syphilis. However, because the test cannot distinguish active infection from past infection, health programs should incorporate confirmatory steps (such as RPR titer testing or novel adjunct assays) and interpret results in the patient’s clinical context. Reinfections are common in high-risk populations and managing them requires sustained efforts in partner management and possibly repeated screening. Additionally, we note the lack of a “perfect standalone test” for syphilis staging, which is why follow-up (either serologically or clinically) is important. For instance, a patient treated should have a falling RPR; we note our study didn’t track that longitudinally, but we refer to known benchmarks for titer decline and reinfection detection [17]. Looking ahead, emerging diagnostics (like IgA-based POCTs) offer hope for more precise identification of active syphilis [13]. Until those become widely available, the judicious use of existing rapid tests like iStatis, in alignment with WHO guidelines [7, 10], will be key to optimizing syphilis control in South Africa and similar settings.

## Data Availability

All relevant data supporting the study findings are in the manuscript and its supplementary materials. Any additional data can be made available if warranted by the corresponding author.

## Abbreviations

ANC: Antenatal clinic
Ab: Antibody
ASSURED: Affordable, sensitive, specific, user-friendly, rapid & robust, equipment-free, and deliverable
AUC: Area under curve
CI: Confidence interval
DNA: Deoxyribonucleic acid
EDTA: Ethylenediaminetetraacetic acid
EIA: Enzyme-linked immunosorbent assay
HIV: Human Immunodeficiency Virus
KZN: KwaZulu-Natal
MSM: Men who have sex with men
NPV: Negative predictive value
PCR: Polymerase chain reaction
POCT: Point-of-care test
PPV: Positive predictive value
ROC: Receiver operating characteristic
RPR: Rapid plasma reagin
SA: South Africa
STI: Sexually transmitted infection
TP: Treponema pallidum
TPHA: T. pallidum hemagglutination
TPPA: T. pallidum particle agglutination
VDRL: Venereal disease research laboratory
WHO: World Health Organization

## References

[1] Kenyon CR, Osbak K, Tsoumanis A. The global epidemiology of syphilis in the past century–a systematic review based on antenatal syphilis prevalence. PLoS Negl Trop Dis. 2016;10(5):e0004711. 27167068 10.1371/journal.pntd.0004711 PMC4864207

[2] Dlamini NR, Phili R, Connolly C. Evaluation of rapid syphilis tests in KwaZulu-Natal. J Clin Lab Anal. 2014;28(1):77–81. 24395488 10.1002/jcla.21647 PMC6807345

[3] Clark M, Grobelna A. Rapid syphilis testing. Can J Health Technol. 2022. 10.51731/cjht.2022.489. 38620374

[4] Gilbert L, Dear N, Esber A, Iroezindu M, Bahemana E, Kibuuka H, Owuoth J, Maswai J, Crowell TA, Polyak CS, Ake JA. Prevalence and risk factors associated with HIV and syphilis co-infection in the African cohort study: a cross-sectional study. BMC Infect Dis. 2021;21:1–7. 34717564 10.1186/s12879-021-06668-6 PMC8557019

[5] World Health Organization. WHO guideline on syphilis screening and treatment for pregnant women. InWHO guideline on syphilis screening and treatment for pregnant women 2017. 29757595

[6] Sidana R, Mangala HC, Murugesh SB, Ravindra K. Prozone phenomenon in secondary syphilis. Indian J Sex Transm Dis AIDS. 2011;32(1):47–9. 21799578 10.4103/2589-0557.81256 PMC3139290

[7] Peeling RW, Holmes KK, Mabey D, Ronald A. Rapid tests for sexually transmitted infections (STIs): the way forward. Sex Transm Infect. 2006;82(suppl 5):v1–6. 17151023 10.1136/sti.2006.024265 PMC2563912

[8] Mabey DC, Sollis KA, Kelly HA, Benzaken AS, Bitarakwate E, Changalucha J, Chen XS, Yin YP, Garcia PJ, Strasser S, Chintu N. Point-of-care tests to strengthen health systems and save newborn lives: the case of syphilis. PLoS Med. 2012;9(6):e1001233. 22719229 10.1371/journal.pmed.1001233 PMC3373627

[9] Bissessor M. Clinical education: Syphilis. Melbourne Sexual Health Centre. 2021 Mar.

[10] Centers for Disease Control and Prevention. Sexually Transmitted Infections Treatment Guidelines. 2021: Syphilis During Pregnancy.

[11] Illinois Department of Public Health. Increasing Early Syphilis Cases in Illinois– Syphilis Staging and Treatment, 2018 Mar.

[12] World Health Organization. WHO guidelines for the treatment of Treponema pallidum (syphilis). InWHO guidelines for the treatment of Treponema pallidum (syphilis) 2016. 27631046

[13] Pham MD, Wise A, Garcia ML, Van H, Zheng S, Mohamed Y, Han Y, Wei WH, Yin YP, Chen XS, Dimech W. Improving the coverage and accuracy of syphilis testing: the development of a novel rapid, point-of-care test for confirmatory testing of active syphilis infection and its early evaluation in China and South Africa. EClinicalMedicine. 2020;24:100440. 32637904 10.1016/j.eclinm.2020.100440 PMC7327895

[14] Papp JR. CDC laboratory recommendations for syphilis testing, united states, 2024. MMWR Recomm Rep. 2024;73:1. 38319847 10.15585/mmwr.rr7301a1 PMC10849099

[15] Wu MY, Gong HZ, Hu KR, Zheng HY, Wan X, Li J. Effect of syphilis infection on HIV acquisition: a systematic review and meta-analysis. Sex Transm Infect. 2021;97(7):525–33. 33219164 10.1136/sextrans-2020-054706 PMC8543214

[16] World Health Organization. Global sexually transmitted infections programme. Dual HIV/syphilis rapid diagnostic tests; 2025.

[17] Centers for Disease Control (CDC). Guidelines for the prevention and control of congenital syphilis. MMWR Supplements. 1988;37(1):1–3. 3123914

